# Supplementary material for: The Proportion of Occupationally Related Cholangiocarcinoma: A Tertiary Hospital Study in Northeastern Thailand
Source: Cancers (Basel). 2022 May 12;14(10):2386. doi: 10.3390/cancers14102386 (PMC9139931; doi:10.3390/cancers14102386)
Supplement: Supplementary file 1 [file cancers-14-02386-s001.zip › cancers-1686072-supplementary.pdf]

Supplementary Materials

The Proportion of Occupationally Related Cholangiocarcinoma: A Tertiary Hospital Study in Northeastern Thailand

Supplementary S1. Questionnaires & case record form: the proportion of occupationally-related cholangio-carcinoma: a tertiary hospital study in northeastern Thailand.

Participant ID ……………………………..Sex…..…………Age……… Date ……………………………….
Start interview time ………………………………… Finish interview time…………………………………

Section 1: Demographic Information Form

(1.1) Date of Birth ………..…………………………………………………………………………….
(1.2) Place of resident Sub-district…………District…………. Province………Region……………….
(1.3) Place of resident (past) Sub-district…………District…………. Province………..Region……………
(1.4) Being ever had found to be infected by liver fluke

- ☐ Never test
- ☐ Tested but negative, please specify Urine ☐ Feces ☐ Blood
- ☐ Tested and positive, , please specify Urine ☐ Feces ☐ Blood
- ☐ Cannot remember

(1.5) Number of occasions using anthelmintics

- ☐ 0
- ☐ 1
- ☐ 2
- ☐ 3
- ☐ More than 3
- ☐ Cannot remember

(1.6) Cigarette smoking

- ☐ Never
- ☐ Ever

(1.7) Alcohol drinking

- ☐ Never
- ☐ Ever

(1.8) Relatives diagnosed with cholangiocarcinoma

- ☐ None
- ☐ Yes, please specify…………………………

(1.9) Being ever had eaten raw fish

- ☐ No
- ☐ Yes

(1.10) Medical condition

- ☐ None
- ☐ Yes (please give detail)
  - ☐Hepatitis B infection
  - ☐Hepatitis C infection

☐Primary Sclerosing Cholangitis
☐Fibro polycystic liver disease
☐Biliary stone
☐Cirrhosis
☐ Other please specify…………..

(1.11) History of thorotrastic exposure

- ☐ No
- ☐ Yes

Section 2: A Form for Collecting Data from a Patient Medical Record and Occupational History

(2.1) Occupational history recorded by treating physicians (data obtained from medical record)

- ☐ One job title
- ☐ Two job titles
- ☐ Three job titles
- ☐ Not available

(2.2) Diagnosis date …………………………………………… (data obtained from medical record**)**

(2.3 Tumor site (data obtained from medical record)

- ☐ Intrahepatic. CCA
- ☐ Extrahepatic CCA

(2.4) Age starting at work ………………… years (A.D. ………..)

(2.5) Occupation (s)

- ☐ 1
- ☐ 2
- ☐ 3
- ☐ More than 3

(2.6) Job title (lifelong)

☐	Printing worker	working period ………….. to ………………
☐	Film production worker	working period ………….. to ………………
☐	Chemical industry (e.g., form, plastic)	working period ………….. to ………………
☐	Textile worker	working period ………….. to ………………
☐	Motor vehicle mechanic	working period ………….. to ………………
☐	Carpenter	working period ………….. to ………………
☐	Painter	working period ………….. to ………………
☐	Welder	working period ………….. to ………………
☐	Roofing worker	working period ………….. to ………………
☐	Ceramic pottery	working period ………….. to ………………
☐	Construction worker	working period ………….. to ………………
☐	Agriculturist	working period ………….. to ………………
☐	Civil servant	working period ………….. to ………………
☐	Road worker	working period ………….. to ………………
☐	…………………………………	working period ………….. to ………………
☐	…………………………………	working period ………….. to ………………
☐	…………………………………	working period ………….. to ………………

(2.7) Occupation classified by major group of ISCO-68

1………………………………………	working period ………….. to ………………
2………………………………………	working period ………….. to ………………
3………………………………………	working period ………….. to ………………
4………………………………………	working period ………….. to ………………

(2.8) Work status

☐ Working

☐ Retired

(2.9) Current or last occupation

Job title ……………………………….. working period ………….. to ………………

(2.10) Working day……………………………… (day(s)/week)

(2.11) Working duration ………………………… (hour(s)/day)

(2.12) Shift work

☐ No (go to 2.14)

☐ Yes

(2.13) Characteristic of shift work

…………………………………………………………………………………………………

(2.14) Job task (job description, chemical hazard, duration, and frequency)

…………………………………………………………………………………………………

(2.15) Hobby

☐ No (go to section 3)

☐ Yes, please specify ………………………………..

(2.16) Hobby description (hobby, chemical hazard, duration, and frequency)

Section 3: Specific Chemical Exposed in Work

(3.1) Chemical/dust exposed in. work

☐ 1,2-Dichloropropane, propylene chloride

☐ Dichloromethane, methylene chloride

☐ Asbestos fiber

☐ Other

☐ No

☐ Yes

☐Can specify chemical's name,
please specify……………...

☐**Cannot** specify chemical's name,
Please give detail ……………………………………………………………….

If participant used to expose these chemicals in 4.1, please interview all chemical/dust that were exposed in 4.2-4.7

(3.2) Procedure of work that exposed these chemical ……………………………………………

(3.3) Frequency that participant exposed. the chemical/dust……………time(s)/week

(3.4) Duration that participant exposed the chemical/dust……………hour(s)/time

(3.5) Respiratory protection equipment using when exposed the chemical/dust

☐ None

☐ Yes, please specify……………………………………………………………………………..

☐ Always, using respiratory protection equipment when exposed the chemical/dust

☐ Sometime, using respiratory protection equipment when exposed the chemical/dust

(3.6) Personal Protective Equipment (PPE) using when exposed the chemical/dust

☐ None

☐ Yes, please specify……………………………………………………………………………..

☐ Always, using PPE when exposed the chemical/dust

☐ Sometime, using PPE when exposed the chemical/dust

(3.7) History of cholangiocarcinoma in co-worker

☐ None

☐ Yes, please specify……………………………………………………………………………..

* If participants suggested to exposure to 1,2-Dichloropropane, dichloromethane or asbestos fiber, please give detail about latency period ………..month (s)………year (s)
